# An exploratory analysis of PM_2.5_/PM_10_ ratio during spring 2016–2018 in Metropolitan Lima

**DOI:** 10.1038/s41598-024-59831-9

**Published:** 2024-04-23

**Authors:** Rita Jaqueline Cabello-Torres, Natalí Carbo-Bustinza, Edison Alessandro Romero-Cabello, Jeremias Macias Ureta Tolentino, Elías A. Torres Armas, Josué E. Turpo-Chaparro, Paulo Canas Rodrigues, Javier Linkolk López-Gonzales

**Affiliations:** 1 Universidad César Vallejo, Research Group ICAMB-UCV, Lima, Peru; 2 Doctorado Interdisciplinario en Ciencias Ambientales, Universidad de Playa Ancha, Valparaíso, Chile; 3 Universidad Nacional Agraria La Molina, Escuela de Ingeniería Ambiental, Lima, Peru; 4 Programa Académico de Ingeniería Ambiental, Facultad de Ingeniería, Universidad de Huánuco, Huánuco, Peru; 5 Instituto de Investigación de Estudios Estadísticos y Control de Calidad, Universidad Nacional Toribio Rodríguez de Mendoza, Chachapoyas, Peru; 6 Escuela de Posgrado, Universidad Peruana Unión, Lima, Peru; 7 Department of Statistics, Federal University of Bahia, Salvador, Brazil

**Keywords:** Ecology, Environmental sciences, Environmental social sciences

## Abstract

Aerosols (PM_2.5_ and PM_10_) represent one of the most critical pollutants due to their negative effects on human health. This research analyzed the relationship of PM and its PM_2.5_/PM_10_ ratios with climatic variables in the austral spring (2016–2018) in Metropolitan Lima. Overall, there was an average PM_2.5_/PM_10_ ratio of 0.33 with fluctuations from 0.30 to 0.35. However, there have also been high point values that reached ratios greater than one. This situation indicates a moderate condition of contamination by particulate matter with a predominance of coarse aerosols in spring, with an increasing trend over the years. The locations *Ate* and *Villa Maria del Triunfo*, especially *Ate*, presented poor quality conditions. Thursdays showed outstanding pollution peaks by PM_10_, and a decrease is visible on Sundays. On the other hand, the PM_2.5_ showed a similar pattern every day, including Sundays. The maximum peaks occurred in the morning and night hours. The increase in anthropogenic emissions associated with the formation of secondary aerosols has been evident, being the case of the location *Campo de Marte*, the one that had a significant increase in ratios PM_2.5_/PM_10_, which confirms a greater intensity of secondary formations of carbonaceous particles from industrial oil sources, vehicle exhaust, as well as aerosols from metal smelting and biomass burning. There were negative correlations of the ratios with PM_10_, temperature, wind speed, and direction, and positive correlations with PM_2.5_ and relative humidity. Contour lines were successfully developed that demonstrated the interaction of climate with PM_2.5_/PM_10_ ratios. This will deepen the exploration of emission sources and modeling, which allows for optimizing air quality indices to control emissions and adequately manage air quality in Metropolitan Lima.

## Introduction

Deteriorating air quality has been linked to respiratory and circulatory problems experienced by residents^1^. It is estimated that each year, exposure to air pollution causes 7 million premature deaths and result in the loss of millions more healthy years of life^2^. Fine particles penetrate directly through the respiratory tract, increasing the risk of mortality over time. This not only causes infections but also promotes lung cancer, diseases such as asthma and cardiovascular diseases^2,3^. Arregocés et al. predicted up to 1.95% mortality due to prolonged exposure to PM_2.5_ for regions of Colombia favoured by winds from the industrial zone^4^. According to Han et al., there is a synergistic effect between pollution sources and meteorological variables, such as temperature, relative humidity, wind speed and intensity on a seasonal basis that influences the contributions to mineral aerosols^5^. Likewise, topographic and land use factors are added, coastal areas are subject to the permanent influence of the marine flow and the continental breeze, masses of marine air that transport coarse sea salt to the continental part during the day^6^. Zhang et al., studied the sources and contributions of PAHs linked to the PM_2.5_/PM_10_ ratio as an indicator of air pollution for the winter and summer periods in Hotan (China)^7^. Zhang et al., identified vehicle emissions, coal combustion and biomass as the main emissions from local sources whose local trajectory is favoured by air masses during winter^7^. The study of PM_2.5_/PM_10_ fractions helps to understand the physical and chemical characteristics of aerosols and natural or anthropogenic emission sources. This research helps to evaluate their spatiotemporal distribution and the effects they have on the environment and human health^8^. In China, PM_2.5_/PM_10_ ratios > 0.5 are related to anthropogenic sources, coming from coal energy consumption or heavy industries that contribute higher proportions of PM_2.5_^9^. Franzin et al., calculated ratios of 0.33 to 0.47 for the city of Sao Paulo and related them to agricultural and industrial activities^10^. Likewise, the use of PM_2.5_/PM_10_ ratios has demonstrated the effectiveness of air quality management or the lack of control when increasing annual trends in the ratios are evident^11^; indicating the increase in anthropogenic emissions^6^. Therefore, it is important to deepen research to understand the relationship that exists between the sources of particulate matter emissions and the climate; explain the relationship between PM_10_ and PM_2.5_ in an urban environment subject to diverse microclimates, geographies, populations and industries, among others^3^.

Air pollution in the urban areas of Lima (Peru) is fundamentally due to the vehicle fleet, the open burning of biomass, the poor disposal of solid waste, and industrial emissions^12^. The city of metropolitan Lima has a population of ten million inhabitants^13^; this is considered one of the most polluted cities in Latin America^14^. Population growth coincides with the increase in air pollution^15^. Among the main polluting agents are PM_10_ (less than 10 microns) and PM_2.5_ (less than 2.5 microns) particles, which exert negative effects on human health^16,17^. The concentrations of these aerosols vary according to the seasonality of the year depending on meteorological conditions^18^. Steenland et al., studied the effect of maximum ambient temperatures associated with PM_2.5_ on cardiorespiratory mortality of patients admitted to emergency rooms in Lima^19^. Velasquez et al., reported 2300 premature deaths from particulate matter in Lima and almost 3,000 deaths from biomass burning^20^.

This situation reflects a serious PM pollution problem and the Peruvian state maintains the Air Quality Monitoring Network in the Lima—Callao Metropolitan Area, developed by *Servicio Nacional de Meteorología e Hidrología del Perú* (SENAMHI). The institution reported from 2016 to 2018 an increase in PM_2.5_ and PM_10_ values, especially during hot periods and stable weather conditions^21^. This extensive network includes three key urban districts such as *Ate* (located in Lima Este), *Villa María del Triunfo* (VMT) (located in Lima Sur) and *Campo de Marte* (CDM) (located in the centre of Lima). These districts were home to 1 million 200 thousand inhabitants in 2018. Its location and topography is associated with very varied micro-climates, as well as a growing demographic expansion, extensive commercial demand, internal demand and transportation. Likewise, there have been incidents of air pollution that have worsened the situation, which is favoured by the stability of weather conditions and emissions from the vehicle fleet. The National Information System for Disaster Prevention and Attention recorded 2,924 fire incidents from 1993 to 2018 only in Lima, followed by Ate (334 reports), VMT (125 reports) and to a lesser extent *Jesús María* (25 reports)^22^.

A practical way to evaluate air quality and emission sources is the application of the PM_2.5_/PM_10_ ratio because it can provide a relevant source of information on the causes of pollution. The processes that develop and affect the atmospheric environment. When the PM_2.5_/PM_10_ ratio is low < 0.5, the predominance of course PM_10_ particles from natural sources is considered^23^. However, if the proportion is high then the emission sources are anthropogenic^24^. Some researchers have demonstrated the relationship of PM_10_ and PM_2.5_ with meteorological variables in certain areas of Lima^12,18,25^, but the PM_2.5_/PM_10_ relationship that exists between both polluting agents in the meteorological conditions of Lima has not yet been studied. Previously, Pereira et al. demonstrated that in CDM, PM_10_ were made up of organic and elemental carbon, monosaccharide, PAHs, oxy- and nitro-PAHs, water-soluble ions; and Ni that comes from metal smelting and oil combustion sources. The researcher also demonstrated that the presence of marine and vehicular aerosols in an area of green areas was due to the environmental transport of contaminants from secondary formations^26^.

The objective of this research is to provide an updated environmental management instrument based on the PM_2.5_, PM_10_, PM_2.5_, PM_10_ relationship and meteorological variables using historical data from three key districts of Metropolitan Lima located in strategic locations of the great megacity. The main contributions of this study are described:

- Application of air quality standards, current national air quality indexes and world health organization guidelines on PM_2.5_ and PM_10_ levels to assess the health risk status of inhabitants of metropolitan Lima.
- Evaluation of the spatio-temporal variability of aerosols using the PM_2.5_/PM_10_ indicator in synergy with PM_2.5_, PM_10_ and meteorological variables to identify pollution sources and the seasonal influence of austral spring (2016–2018).
- Implementation of the analysis of dispersion, correlational, and contour lines to demonstrate the interaction between meteorological variables and PM_2.5_/PM_10_ ratios by identifying emission sources.

The rest of the paper is organized as follows. Sections presents the “Methods” that detail the methodology applied in evaluating the ratios, relations with the meteorological variables, and identification of the emission sources. Then, Section outlines show the “Results and discussion”, which details the relevant research findings and discusses the theoretical approaches and historical background of other studies. Finally, Sections provide the “Conclusions” of this study, with recommendations for future research related to the application of indices based on PM_2.5_/PM_10_ ratios.

## Methods

### Study area

**Figure 1:**
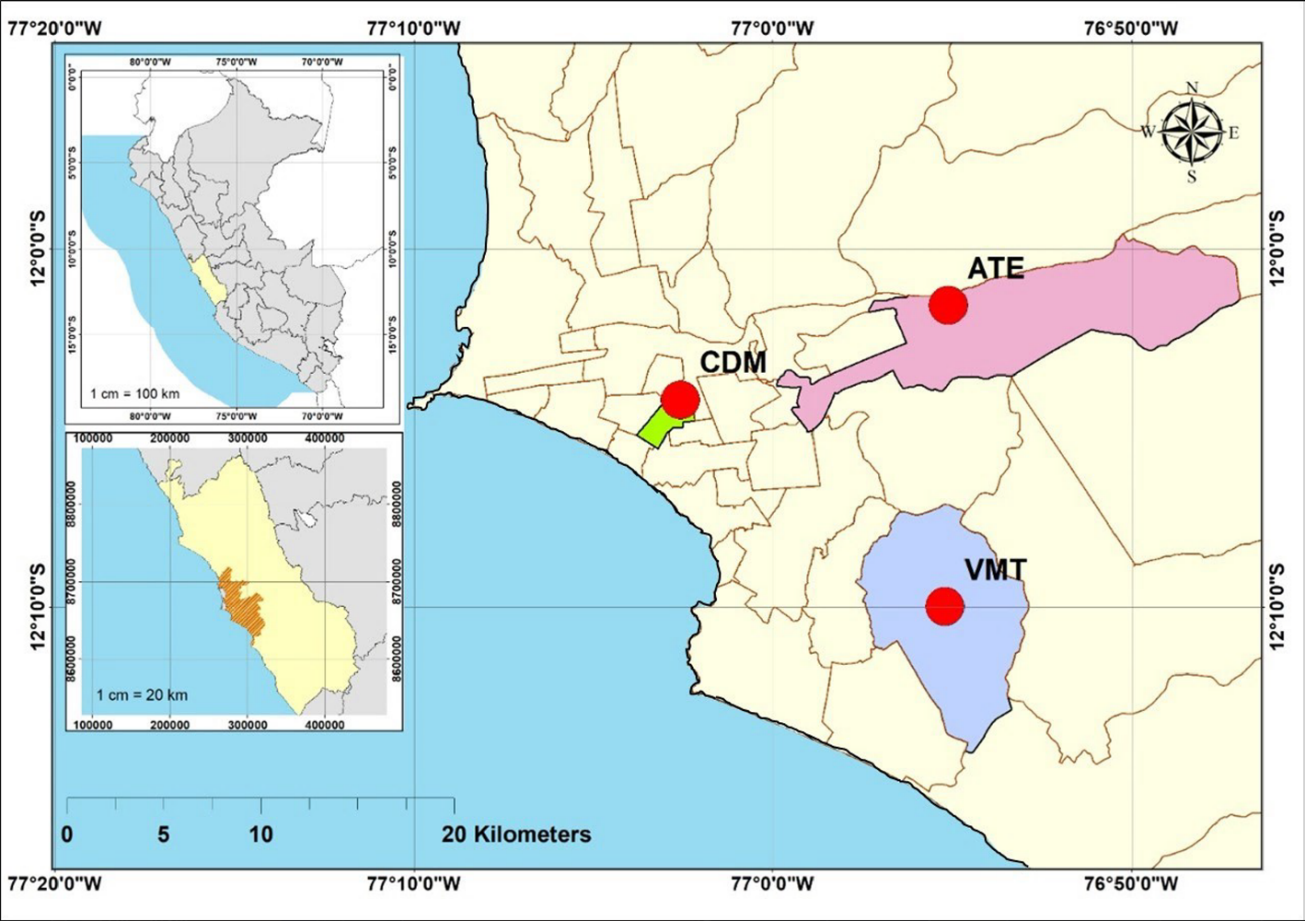
Position chart of the PM_2.5_, PM_10_ and meteorological variables sampling sites that are part of the SENAMHI Monitoring Network in Metropolitan Lima (Peru). Austral spring: 2016–2018. This map was created using ArcGIS Desktop 10.8.x. https://www.esri.com/en-us/arcgis/products/arcgis-desktop/resources.

The characteristics of PM_2.5_ and PM_10_ and their ratios (PM_2.5_/PM_10_) have been investigated in three districts of the Metropolitan network of Lima (Peru). These districts are located in strategic places in Lima: Ate, VMT and CDM. These are subject to different microclimates and a population of approximately one million inhabitants. The monitoring sites are classified as places of intense urban traffic, however CDM is a fully paved commercial area with the presence of a green area, Ate is further from the coast, has industries and less paved land, it is a district adjacent to the central highway where more than 7 thousand vehicles circulate daily towards the interior of the country. On the other hand, VMT is desert in nature, with sandy and poorly paved soils, located near the South Pan-American Highway, one of the main roads with intense national and international traffic. The CDM station is located in the *Jesús María* District at 117 meters above sea level with coordinates: 12^∘^4ʹ14.03″ AL—77^∘^2ʹ35.3″ WL. The Ate station is located in the district of the same name at 362 meters above sea level with coordinates: 12^∘^1ʹ34″ SL—76^∘^55ʹ7″ WL and the VMT District station is located at 272 meters above sea level with coordinates: 12^∘^9ʹ59.01″ SL and 76^∘^55ʹ11.99″ WL. Figure 1 shows the position chart used in this research, during the austral spring 2016–2018.

### Air quality monitoring stations and data collection

The monitoring period covered the austral spring season (September 22 to December 22) from 2016 to 2018. Data on PM_2.5_ and PM_10_ concentrations, ambient temperature (°C), relative humidity (RH, %) have been processed, wind speed (WS; m/s) and wind direction (WD; degrees), the data have been provided by the SENAMHI Air Quality and Meteorology Monitoring network. Only the VMT site does not have WS and WD data. The research has considered the comparison of PM_2.5_ and PM_10_ data with the reference values of the WHO Guide^27^ for PM_2.5_ (10 $\upmu$g/m^3^ annual average; 25 $\upmu$g/m^3^ average of 24 hours) and PM_10_ (20 $\upmu$g/m^3^ annual average and 50 $\upmu$g/m^3^ 24-hour average). Additionally, PM data have been compared to current national air quality standards (NAAQS: PM_2.5_=50 $\upmu$g/m^3^; PM_10_=100 $\upmu$g/m^3^) and National Air Quality Indices (NAQI). The NAQI is calculated using the following expressions:

$$\begin{aligned} \textrm{I}(\textrm{PM}_{10})=[\textrm{PM}_{10}]*100/150 \\ \textrm{I}(\textrm{PM}_{2.5})=[\textrm{PM}_{2.5}]*100/25 \end{aligned}$$

where: I(PM_10_) and I(PM_2.5_) express the calculated NAAQI and the value inside the parentheses corresponds to the observed PM (PM_10_, PM_2.5_). Table 1 shows the national NAAQI classification. Furthermore, the application of the PM_2.5_/PM_10_ ratios has been evaluated as a new air quality management instrument that can be implemented to optimize pollution control and the identification of the main emitting sources of PM_2.5_ and PM_10_.

### Statistical analysis

In this research, 124,488 data provided by SENAMHI have been analysed. This amount of information corresponds to 90.5% of the data on PM_2.5_, PM_10_ and meteorological parameters (T, HR, WS, WD), since VMT does not have WS and WD data. To process the information, the R Studio program was used and to analyse the descriptive and inferential statistics of the PM and climate data, the Pearson correlation coefficient and a multivariate Gaussian regression analysis were applied considering the p-value for analyse the level of significance (p < 0.05). The dispersion of the PM_2.5_/PM_10_ ratios was analysed and contour isolines with Z axis were applied to analyse the bivariate effect of meteorological conditions on the PM_2.5_/PM_10_ ratio (supplement). Python’s Windrose package was used to show how wind speed and direction are normally distributed at a given location.

## Results and discussion

### Concentrations of PM_2.5_, PM_10_, PM_2.5_/PM_10_ ratios and meteorological variables during the austral spring

Table 1 presents a general statistical summary of the concentrations of particulate matter (PM_2.5_ and PM_10_), meteorological variables and PM_2.5_/PM_10_ ratios recorded during the austral spring 2016-2018 (September 22 to December 22) in the three sampling sites Ate, VMT and CDM.

**Table 1:** Descriptive statistics that summarize the general meteorological conditions and particulate matter (PM_10_ and PM_2.5_) recorded from 2016 to 2018 (austral spring) in Ate, VMT and CDM.

MS	SP	PM_2.5_	PM_10_	PM_2.5_/PM_10_	T(^∘^C)	RH	WS	WD
ATE	Mean ± sd	39 ± 22	118 ± 47	0.35 ± 0.23	18 ± 3	81 ± 10	1 ± 1.0	243 ± 32
	Median	34	112	0.32	18	84	1	244
	Min	3	6	0.01	2	47	0	26
	Max	262.4	503.9	5.53	26.55	99.5	3.63	327.8
	% value > NAAQS/WHO	24/71	62/97					
VMT	Mean ± sd	24 ± 14	78 ± 32	0.33 ± 0.20	19 ± 3	87 ± 10		
	Median	21	73	0.29	19	90		
	Min	3	8	0.03	13	54		
	Max	164.2	291	3.29	29.41	99.8		
	% value > NAAQS/WHO	5.8/37	20/55					
CDM	Mean ± sd	15 ± 8	58 ± 30	0.30 ± 0.21	18 ± 2	83 ± 7	3 ± 1.0	217 ± 28
	Median	14	52	0.27	18	84	3	215
	Min	3	6	0.01	14	42	0	2
	Max	60.8	391.1	2.92	30.7	97	8.5	359
	% value > NAAQS/WHO	0.26/11	5.5/55					

National Ambient Air Quality Standards (NAAQS) of Peru (50 $\upmu$g/m^3^ for PM_2.5_ and 100 $\upmu$g/m^3^ for PM_10_). T=atmospheric temperature (^∘^C); RH=relative humidity (%); WS=wind speed (m/s); WD=wind direction (degrees).

The average concentrations of PM_2.5_ during 2016-2018 fluctuated from 3 to 262.4 $\upmu$g/m^3^ (Ate) and for PM_10_ the value ranged from 6 to 503.90 $\upmu$g/m^3^ (Ate). Ate has stood out for maintaining the maximum PM values, compared to the other sites: Ate > VMT > CDM. Table 1 also shows the percentage of PM values above the NAAQS. Regarding PM_10_, this sequence has been recorded: Ate (24%) > VMT (6%) > CDM (0.26%), while for PM_2.5_: Ate (62%)> VMT (20%)> CDM (5.5%). On the other hand, 97% of the PM_2.5_ values and 71% of the PM_10_ values in Ate exceeded the WHO guideline values for 24-h measurements, showing a high risk for human health^27^. Likewise, the general average of the PM_2.5_/PM_10_ ratio was 0.33 (range: 0.30 in CDM - 0.35 in Ate). The values were similar to those reported in other regions of the world for warm periods such as in Italy (0.38) considering that this proportion varies seasonally with increases in cold periods^28^. In Bahrain (0.353) where the contribution of PM_2.5_ emissions from densely located local industries stands out^29^. In contrast, ratios > 0.5 have also been reported^7,30^. A PM_2.5_/PM_10_ ratio equal to 0.5 has previously been used as a threshold value for air pollution from PM emission sources^7,31^. In this research, the general average of the PM_2.5_/PM_10_ ratios is less than 0.5, however 10.4% of the ratios > 0.5 followed the following order: Ate (16%, Max: 5.53) > VMT (14%, Max: 3.29) > CDM (11%, Max: 2.92). Previously, Valdivia reported ratios of 0.65 considering the winter periods of greatest pollution^32^.

**Figure 2:**
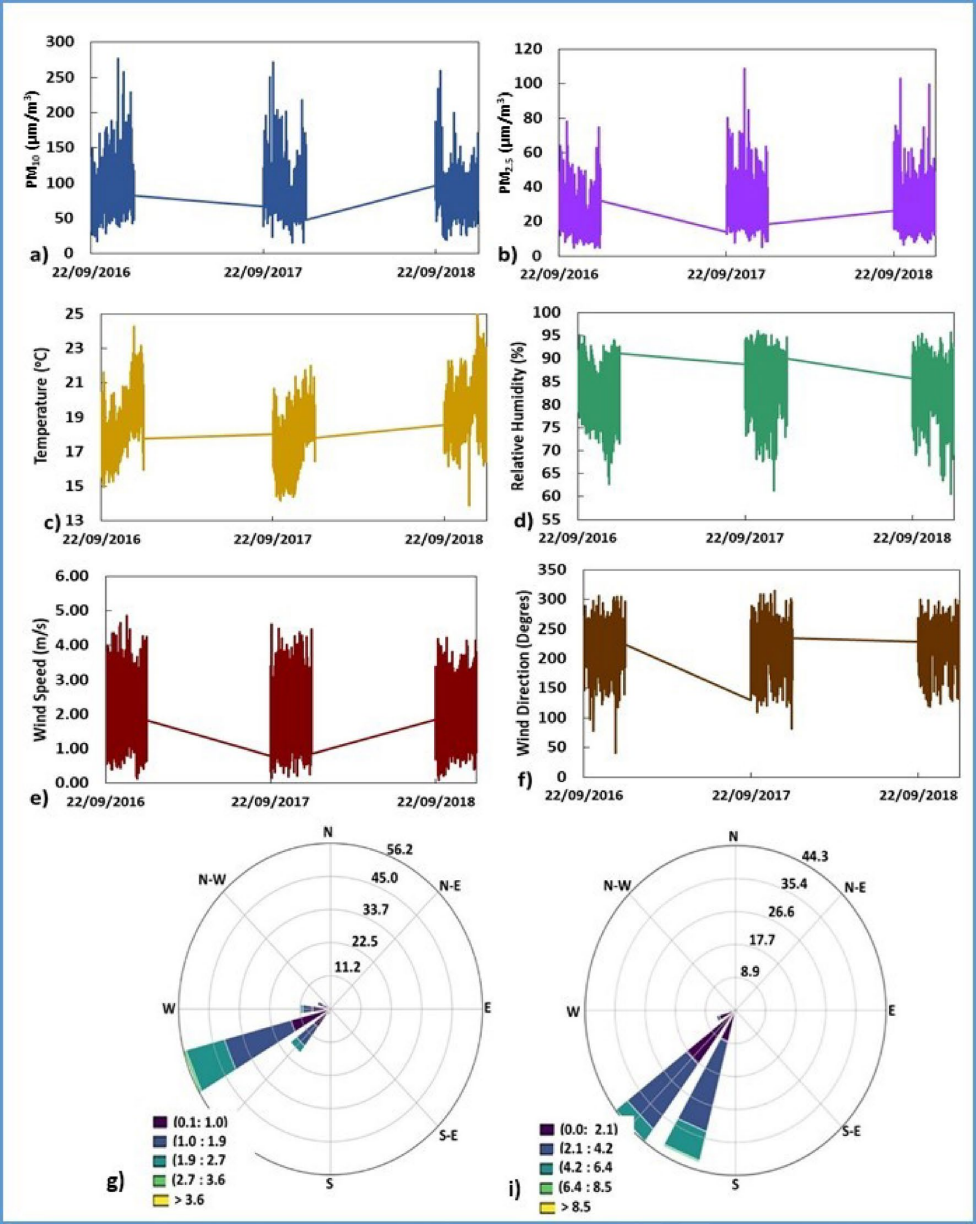
Distribution of PM and meteorological variables per hour: (**a**) PM_10_ ($\upmu$g/m^3^); (**b**) PM_2.5_ ($\upmu$g/m^3^); (**c**) temperature (^∘^C); (**d**) relative humidity (%); (**e**) Wind speed and (**f**) wind direction, (**g**) Wind rose for Ate, (**i**) wind rose for CDM, during the austral spring 2016–2018.

Regarding the meteorological variables, Fig. 2 shows the average values, with a temperature range of 18 to 19 °C and a typical rise from September to December 22 (beginning of summer). The HR was similar for Ate and CDM (81% and 83% respectively) and somewhat higher for VMT (87%). This situation was associated with weak and moderate wind flows (averages < 5 m/s) in the morning, moderate to strong in the afternoon and weak winds at night; due to persistent atmospheric stability that limits vertical dispersion with horizontal transport directions in a northerly, north-easterly direction.

### Interannual distribution of PM_2.5_, PM_10_ and PM_2.5_/PM_10_ ratio during the austral spring (2016–2018)

Table 2 summarizes the interannual results of PM_2.5_ and PM_10_ concentrations during the spring of 2016 to 2018 at the selected sampling sites. The results of the annual averages summarize the percentage of PM>NAAQS concentrations in the following order: (i) PM_10_: Ate (60–65%) > VMT (12–29.5%) > CDM (1.5–10%) (ii) PM_2.5_: Ate (20–24%) > VMT (4–8%) > CDM (0–0.6%). It is important to highlight that Ate is located on both sides of the country’s central highway, where vehicular traffic has increased^33^. Likewise, VMT has a sandy desert soil with a road saturated with public and private transportation that includes large cargo trucks^34^. On the other hand, CDM is located in the district of *Jesús María*, this is a 100% paved district, and is mainly affected by the automobile fleet^35^. It has been shown that sources of air pollution are concentrated in urban areas such that winds transport these pollutants to surrounding areas^36^.

**Table 2:** Statistical description of PM_2.5_, PM_10_ concentrations and PM_2.5_/PM_10_ ratio during the 2016–2018 austral spring season..

MS	Year	SP	PM_10_	PM_2.5_	PM_2.5_/PM_10_
ATE	Springer, 2016	Mean ± sd	114 ± 43	35.02 ± 22.11	0.33 ± 0.19
		Median	109	29	0.27
		Min	15	3	0.01
		Max	503.90	168.10	1.47
		% value > NAAQS	60	20	
	Springer, 2017	Mean ± sd	123 ± 48	42.65 ± 21.24	0.36 ± 0.17
		Median	115.89	38.30	0.34
		Min	16.15	3.90	0.03
		Max	495.90	168.10	2.58
		% value > NAAQS	65	29	
	Springer, 2018	Mean ± sd	115.98 ± 48	38.05 ± 22	0.37 ± 0.3
		Median	109.40	33.70	0.33
		Min	6.41	3.90	0.03
		Max	443.90	262.40	5.53
		% value > NAAQS	62	24	
VMT	Springer, 2016	Mean ± sd	78.24 ± 29.68	22.49 ± 13.76	0.31 ± 0.19
		Median	74.29	19	0.27
		Min	8.27	3.2	0.03
		Max	291	82	1.51
		% value > NAAQS	18	5	
	Springer, 2017	Mean ± sd	86.28 ± 36	27.44 ± 16	0.34 ± 0.21
		Median	79.38	24	0.30
		Min	8.71	3.60	0.04
		Max	274	164.20	3.29
		% value > NAAQS	29.5	8	
	Springer, 2018	Mean ± sd	70.66 ± 26	22.42 ± 13	0.33 ± 0.19
		Median	66.89	19.80	0.30
		Min	14.84	3.30	0.03
		Max	222.50	98.00	2.55
		% value > NAAQS	12	4	
CDM	Springer, 2016	Mean ± sd	65.94 ± 40.97	10.92 ± 6.1	0.2 ± 0.13
		Median	56.8	9.6	0.17
		Min	8.97	3	0.01
		Max	394.1	45.6	1.25
		% value > NAAQS	10	0	
	Springer, 2017	Mean ± sd	55.9 ± 21	17.79 ± 9.3	0.36 ± 0.24
		Median	52.57	15.9	0.31
		Min	6.33	3.30	0.04
		Max	148.4	60.8	2.92
		% value > NAAQS	4.5	0.6	
	Springer, 2018	Mean ± sd	51.64 ± 19.7	16.63 ± 7.3	0.35 ± 0.19
		Median	48.72	15.10	0.32
		Min	6.33	3.10	0.03
		Max	285.80	58.60	2.38
		% value > NAAQS	1.50	0.10	

According to Table 2, in spring the ratios remain from 0.31 to 0.37 except for CDM which in 2016 presented the lowest ratio of all (PM_2.5_/PM_10_ = 0.2), indicating a lower contribution of PMs to the atmosphere.

It is important to highlight those previous studies carried out on the chemical composition of PM_10_ in CDM showed contributions from the sea breeze (sulphates, nitrate, ammonium and chlorides). In addition, to terrestrial contributions from direct vehicle emissions, the resuspension of road dust, metal smelting, oil combustion, oil refineries, PAHs emissions and secondary organic carbon formations, due to combustion of biomass and solid waste^26^.

**Figure 3:**
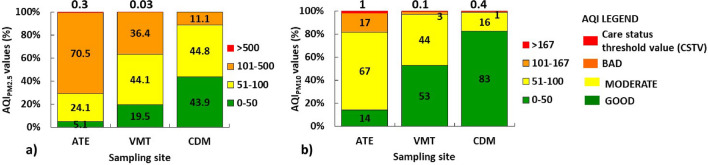
Range of percentages of PM values (interannual) according to the national NAAQI for: (**a**) PM_2.5_ and (**b**) PM_10_.

Regarding the NAAQI calculated for the PM, the results showed the PM_2.5_ levels of poor quality in the following order: Ate (70.5%) > VMT (36.4%) > CDM (11%) and levels of PM_10_ of moderate quality in the following order: Ate (67%) > VMT (44%) > CDM (15%) (Fig. 3). Attempts to optimize air quality management have been related to the 4-year strategic plan (2021–2025), to reduce air pollution within the Lima-Callao Metropolitan Area, through the management of emission sources. This agrees with the National Environmental Policy^37^. However, in practice there are still no positive results for the protection of human health and improvement of quality of life.

### Spatiotemporal variations of PM_2.5_, PM_10_, PM_2.5_/PM_10_ ratio and meteorological variables

**Figure 4:**
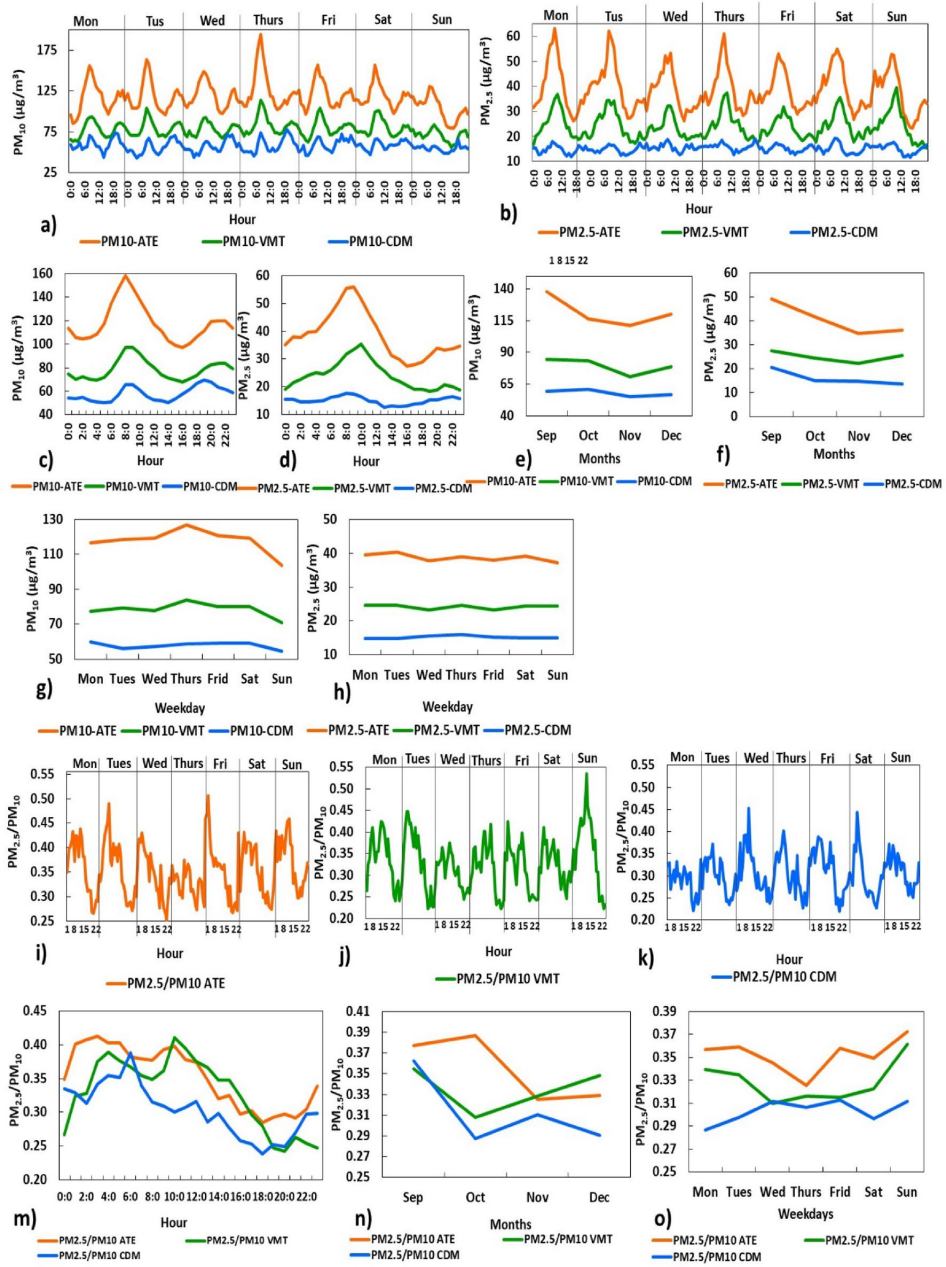
Temporal variations of PM_10_ ($\upmu$g/m^3^), PM_2.5_ ($\upmu$g/m^3^) and their ratios (PM_2.5_/PM_10_) from September to December (2016–2018) in Ate, VMT y CDM of Metropolitan Lima (Peru).

Figure 4 shows the hourly concentrations of PM_10_ during the week, generally showing two very well-marked daily peaks around 08:00 hours and 10:00 hours Ate develops a first peak around 08:30 hours (194.29 $\upmu$g/m^3^) that decreases towards the afternoon (14:00 hours–16:00 hours), due to the increase in wind intensity (maximum: 3.63 m/s). The wind disperses and decreases PM concentrations and then a second peak (maximum: 126.76 $\upmu$g/m^3^) occurs around 22:00 hours as the WS decreases. In VMT the first peak occurs at 07:30 hours until 10:00 hours (maximum: 114.5 $\upmu$g/m^3^). Then the wind disperses the particles around 13:00 hours^38^, until the second peak is formed from 20:00 to 22:00 hours (maximum: 87.14 $\upmu$g/m^3^). For its part, CDM produces peaks at 08:30 hours and 20:00 hours (maximum averages of 74.29 $\upmu$g/m^3^ and 78.17 $\upmu$g/m^3^). The decreases in concentrations are recorded in the early hours of the morning (around 03:00 hours) and in the afternoon (around 16:00 hours), associated with strong winds (maximum 8.5 m/s). This is because part of the coarse material is re-suspended dust, these would be carried by the wind in the early hours of the morning, when atmospheric instability and the greatest vertical and horizontal movement of the wind occur^11^. Aerosols increase during the night due to the lower intensity of the night wind (PM_10_: 69.33 $\upmu$g/m^3^, 19:00 hours).

In relation to PM_2.5_, certain similarities were also shown in the study areas, with an increasing trend from midnight and early morning towards (from 08:00 hours to 10:00 hours). In Ate a peak appears from 08:00 hours to 09:30 hours (maximum: 63.15 $\upmu$g/m^3^), then a decrease in values occurs until 17:00 hours when the new cycle begins. In VMT the maximum peak (39.54 $\upmu$g/m^3^) occurs around 10:00 hours and in CDM at 08:30 hours (maximum: 19.9 $\upmu$g/m^3^), although a second is also observed peak in CDM around 22:00 hours (19.01 $\upmu$g/m^3^) (Fig. 4b–d). Obviously, vehicular traffic activity increases in the morning hours and to a lesser extent after 20:00 hours. The vehicle fleet plays a relevant role in increasing PM concentrations^11^.

On the other hand, the monthly variation (Fig. 4e,f) showed a certain decrease in PM between October and November, favoured by higher relative humidity, and higher wind intensities (in November; Ate: 1.26 m/s; CDM: 2.78 m/s) compared to the months of December^21^. Regarding the days of the week (Fig. 4g,h), these were divided into weekdays (Monday to Friday) and weekends (Saturday and Sunday). In Ate and VMT on Thursdays a peak of higher average concentration of PM_2.5_ develops (127 $\upmu$g/m^3^ and 83.7 $\upmu$g/m^3^ respectively); associated with the supply of goods and services to industries, businesses and urban transportation, while on Sundays the decrease in values is observed. In CDM, a decrease in PM_10_ values was also observed over the weekend (Sunday: 54.6 $\upmu$g/m^3^). These findings reaffirm that the highest PM contents during the day occur in the warmest hours^11^, but spring is an intermediate season where a climate that ascends from cold to warm temperatures can be observed. During this period, the rise of the thermal inversion layer occurs, this favours atmospheric instability, without significant contributions of humidity and increases in ambient temperature dispersing the pollutant^25^.

### Scatter plot, correlation and determination factor

**Figure 5:**
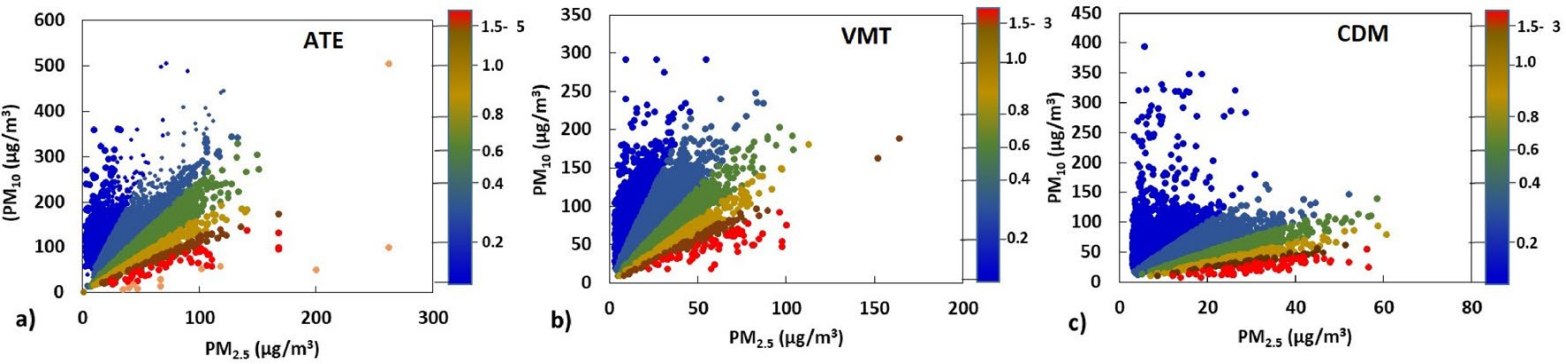
Scatter diagrams of PM_10_ ($\upmu$g/m^3^), PM_2.5_ ($\upmu$g/m^3^) and their ratios (PM_2.5_/PM_10_), from September to December (2016–2018) in Ate, VMT, and CDM.

**Figure 6:**
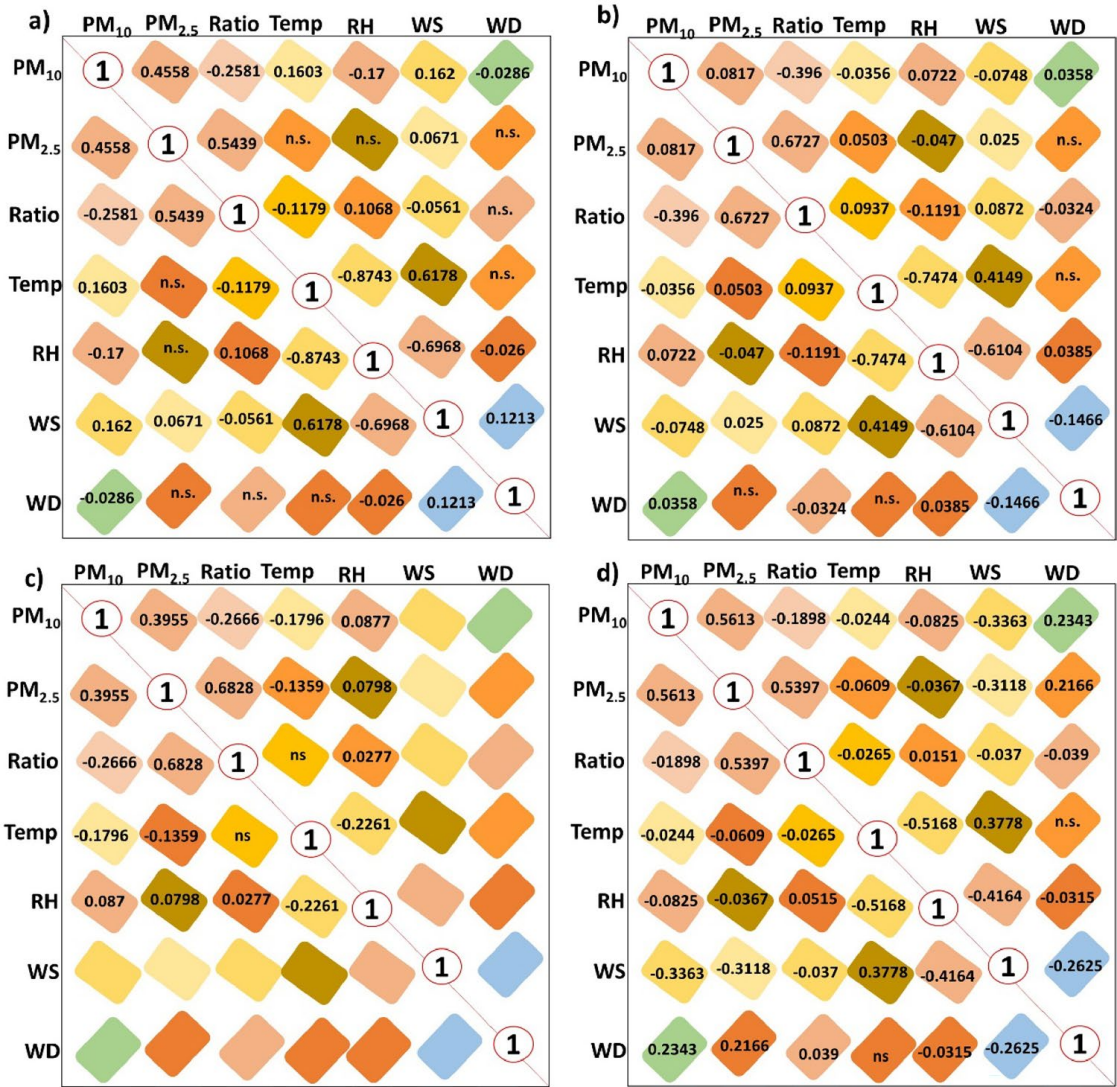
Correlations between PM_10_, PM_2.5_, ratio (PM_2.5_/PM_10_) and the four meteorological variables in (**a**) Ate; (**b**) CDM; (**c**) VMT, and (**d**) General correlation of the 3 sampling stations.

A scatter plot of PM_10_ versus PM_2.5_ at the monitoring sites using hourly data from the austral spring from 2016 to 2018 is shown in Fig. 5. Scatter plots show 7 colours represented by blue for ratios < 0.2, light blue for ratios < 0.4, green for ratios < 0.6, mustard for ratios < 0.8; brown for ratios < 1 and red for ratios > 1, these form fan-shaped distributions. According to Munir et al.^11^, the blue region suggests the predominance of coarse aerosols related to higher concentrations of PM_10_ that include material generated by mechanical routes related to the construction industry. Lima has industries producing non-metallic minerals, cement factories, limestone, dolomite and concrete extraction, especially in Ate and VMT. On the other hand, from beige to red, high values of the PM_2.5_/PM_10_ ratio (>0.8) are observed, these would be composed mainly of carbonaceous species from contributions of fossil fuels, organic carbon from secondary emissions from the burning of biomass. This coincides with what was previously reported by Pereyra et al.^26^ for CDM and coincides with what was found in other cities where direct combustion sources predominate or present pollutants formed in secondary reactions in the atmosphere, sulphate and nitrate ions are also included in this group^11^.

The application of the Pearson correlation statistical method and multivariate Gaussian regression is then evaluated to evaluate the existing relationships. Figure 6 shows the correlation between the PM_10_, PM_2.5_, PM_2.5_/PM_10_ ratio; temperature (°C), relative humidity %), wind speed (m/s), wind direction (degrees) per hour during the austral spring season (2016–2018) in the districts of Ate, VMT, CDM.

Regarding the correlations for PM and meteorological variables, our results disagree with the findings of Sirithian and Thanatrakolsri^3^, for rural and urban cities in Taiwan, since no strong correlations were established for PM and RH/T. The PM_10_-RH/T correlations for Ate are the only consistent values (rPM_10_-T: 0.1603; rPM_10_-RH: – 0.17) and although the other areas did not reflect appreciable correlations and linear adjustments (r < 0.1; R^2^ < 0.03), the correlations observed for T and RH with PM_10_ were always inverse, VMT also showed a weak PM_10_-T correlation (r: – 0.1796). Lima is a very particular place and in this analysis the topographic aspect has not been taken into account. The presence of hills or slopes, of basins (Chillón and Rímac) capable of confining humidity and winds from the sea, promote the appearance of local microclimates that cause chaotic behaviour with atmospheric pollutants^39^. Likewise, the correlation analysis for PM_2.5_ did not produce significant values. Regarding the correlations of the PM_2.5_/PM_10_ ratios with RH and T, the values were inverse, however only Ate and CDM showed weak correlations (Ate rPM_2.5_/PM_10_-T = – 0.1179 and Ate rPM_2.5_/PM_10_-RH = 0.1068; CDM rPM_2.5_/PM_10_-T = 0.0937 and CDM RH-ratio = – 0.1191).

Likewise, wind flows, at the local level only Ate presented a weak correlation for PM_10_-WS (r= 0.162). It is important to highlight that in this research 90.5% of data validated by SENAMHI was used due to the absence of information about WS and WD in VMT. Despite this, at a general level, better negative correlations have been established for PM_10_, PM_2.5_ and WS (rPM_10_-WS: – 0.3363; rPM_2.5_-WS: – 0.3168) and positive correlations for WD (rPM_10_-WD: 0.2343; rPM_2.5_-WD: 0.2166).

This indicates that wind is the variable that maintains a relevant role in the environmental transport, dispersion and removal of PM through dry or humid atmospheric deposition processes. Positive correlations suggest atmospheric transports from non-local sources, while negative correlations would indicate local dust re-suspension. Munir et al.^11^, points out that in arid areas that receive little rain (and this occurs in Lima) high wind speed and temperature promote wind turbulence and the re-suspension of dust and sand particles and increase the concentrations of particles in the atmosphere^40^. These findings indicate that it is possible to relate RH and T with PM_10_ at the local level, and that the PM_2.5_/PM_10_ ratio can be used in Ate and CDM to correlate with RH and T. The method also serves to establish correlations between PM_10_ and PM_2.5_ with the WS and WD at the local level.

**Figure 7:**
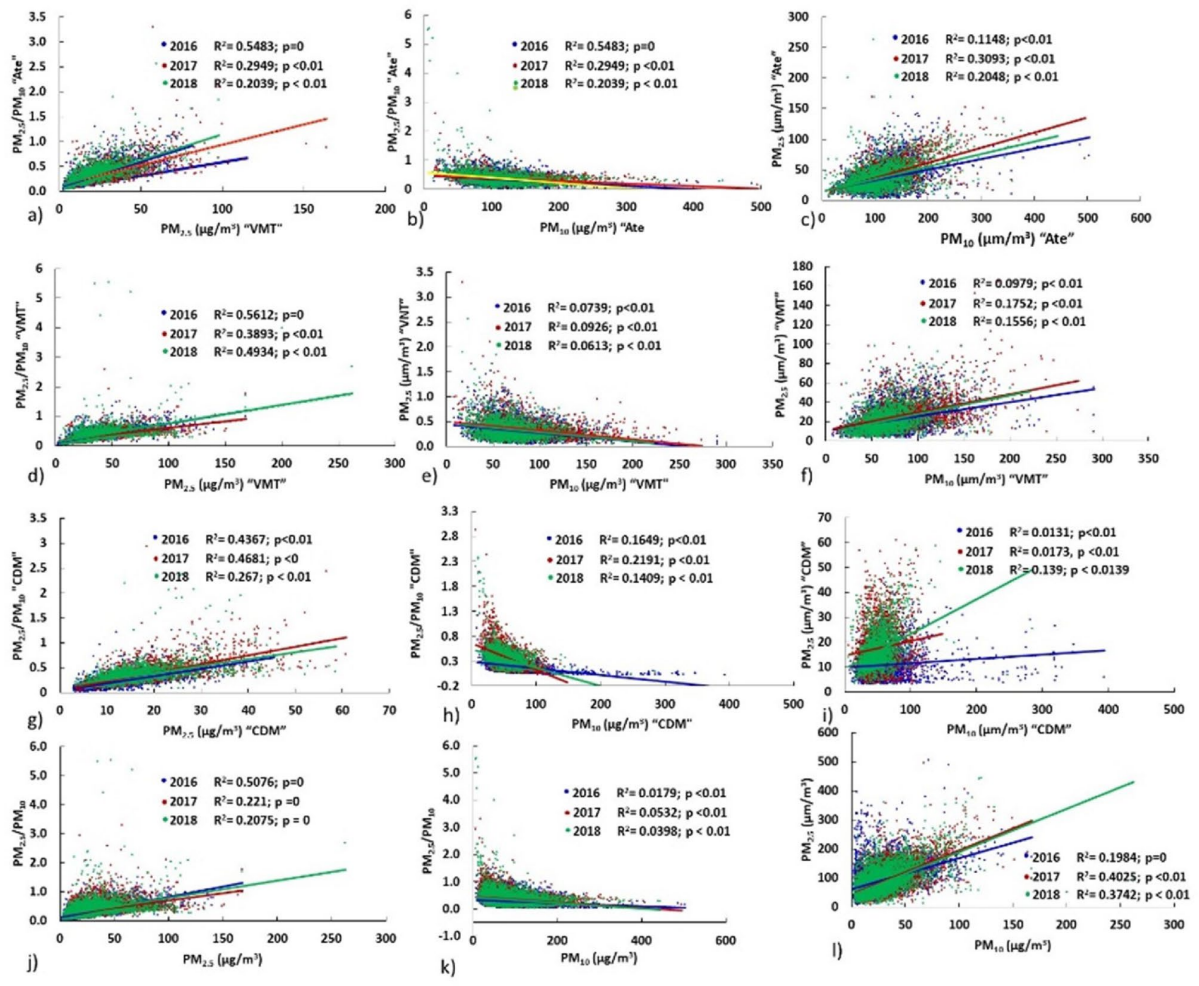
Determination factor for the correlations of PM_10_, PM_2.5_, versus ratio (PM_2.5_/PM_10_) of: (**a**–**c**) Ate; (**b**–**d**) CDM; (**e**–**g**) VMT and (**j**–**l**) general evaluation of the 3 sampling stations.

For PM_10_–PM_2.5_/PM_10_, negative correlations were obtained for the three sampling sites with values that ranged from r= -0.2585 to r= -0.396 (average r: -0.1898). The relationship for PM_2.5_–PM_2.5_/PM_10_ was higher (r: 0.5439 to 0.6828) and presented a direct average correlation coefficient equal to 0.5397. When applying the determination factor R^2^ (Fig. 7) for PM_2.5_ and PM_2.5_/PM_10_, significant and moderate values were produced (R^2^Ate = 0.2039–0.5483; R^2^VMT = 0.3893–0.5612, R^2^CDM = 0.267–0.4367; p < 0.01), the biggest factors corresponded to the 2016.

This finding is important because it establishes in a practical and reliable way the identification of the sources that put the health of the inhabitants of the area at risk. Previously, Pereira et al.^26^, characterized PM_10_ in CDM, however, they warned about the formation of secondary particles in the atmosphere. Likewise, Huyen et al.^41^, pointed out that fine aerosols (PM_2.5_) that include ultrafine PM come from a significant contribution of secondary organic carbon, so the growth of these particles depends on the amount of surface water molecules present in the aerosol (associated to the relative humidity), since relative humidity exerts a different effect on the concentrations of fine and coarse particles^42^. In general, the levels of PM_2.5_ and PM_10_ observed in the southern spring of 2016-2018 were observed in the towns of VMT, CDM and Ate in Metropolitan Lima. To evaluate the health risk status of the inhabitants of the big city, the current standards that regulate air quality in the country, and the WHO guideline values, have been used.

Likewise, the spatiotemporal variability of the particulate material has been evaluated and the correlation coefficient (r) and the determination factor R^2^ have been applied to establish relationships of meteorological variables such as HR, T, WS and WD with the aerosol concentration. With this objective, the PM_2.5_/PM_10_ ratio has been incorporated as a new indicator whose values allow identifying the sources of pollutant emissions, whether primary or secondary. This study has allowed us to better understand the local and macro-level climatic influence on the distribution of pollutants and their sources of pollution. The findings have been supported with previous information from Ilizarbe-Gonzáles et al.^43^, who analysed the physicochemical composition of PM_10_ samples in a district in the north of Lima and in San Juan de Lurigancho (eastern Lima) in autumn 2017 and identified the polluting sources that affect air quality. Also Pereira et al.^26^, analysed the physicochemical composition of PM_10_ particles and the presence of secondary and primary aerosols such as marine aerosols, demonstrating the influence of wind transport on the load of fine material towards CDM. Other complementary studies related to PM modelling^14,20,32,44^ and others related to the epidemiological effect^12,45^ have been reviewed. These indicators with meteorological variables can be applied locally by regional governments and can be applied to evaluate levels of air pollution and risk to human health. Likewise, the sources of polluting emissions can be identified, which will help optimize air quality control and management instruments.

**Figure 8:**
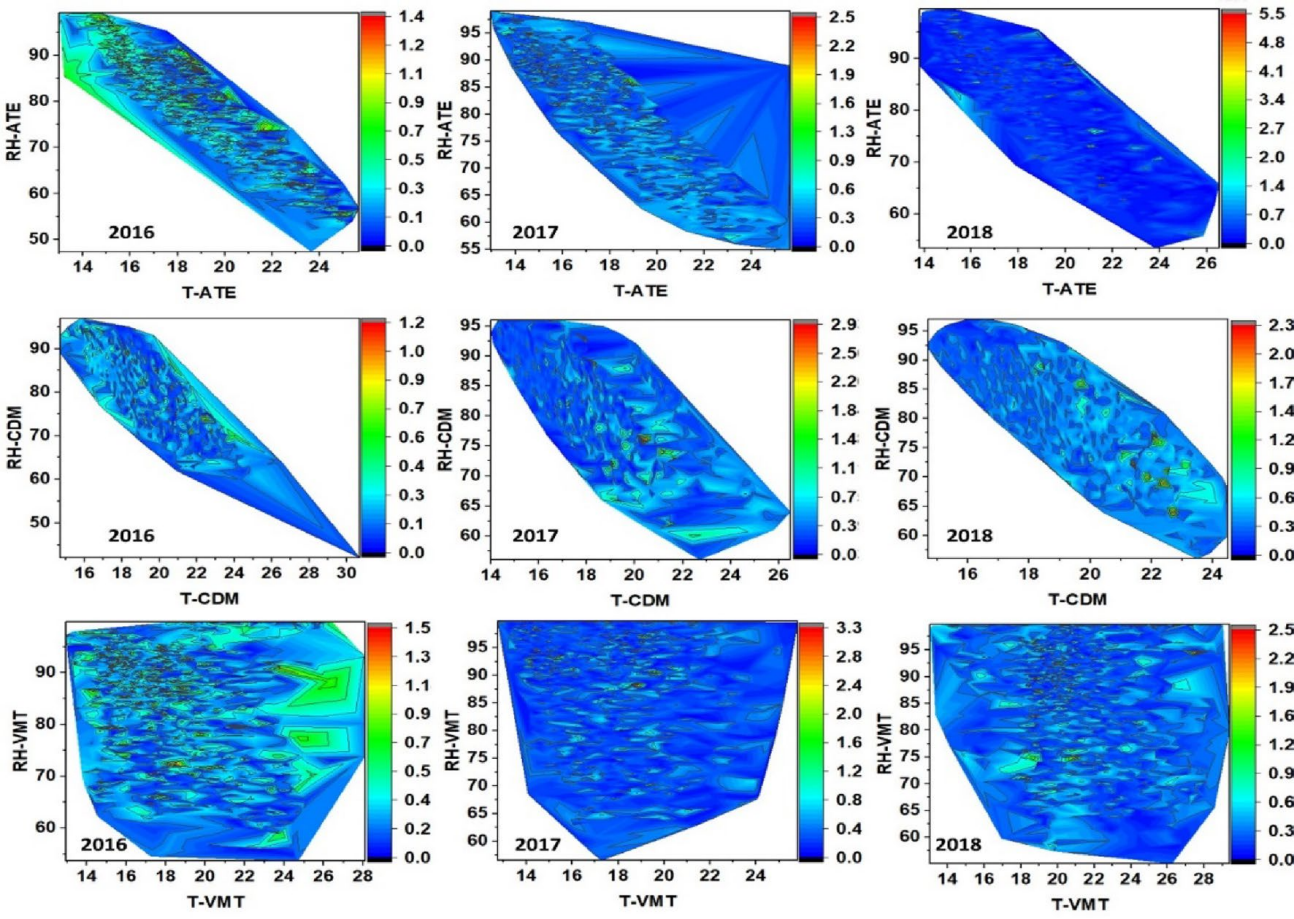
Contour lines for the distribution of (relative humidity–temperature) RH-T-PM_2.5_/PM_10_ ratio in the spring (2016–2018), in Ate, VMT, and CDM.

**Figure 9:**
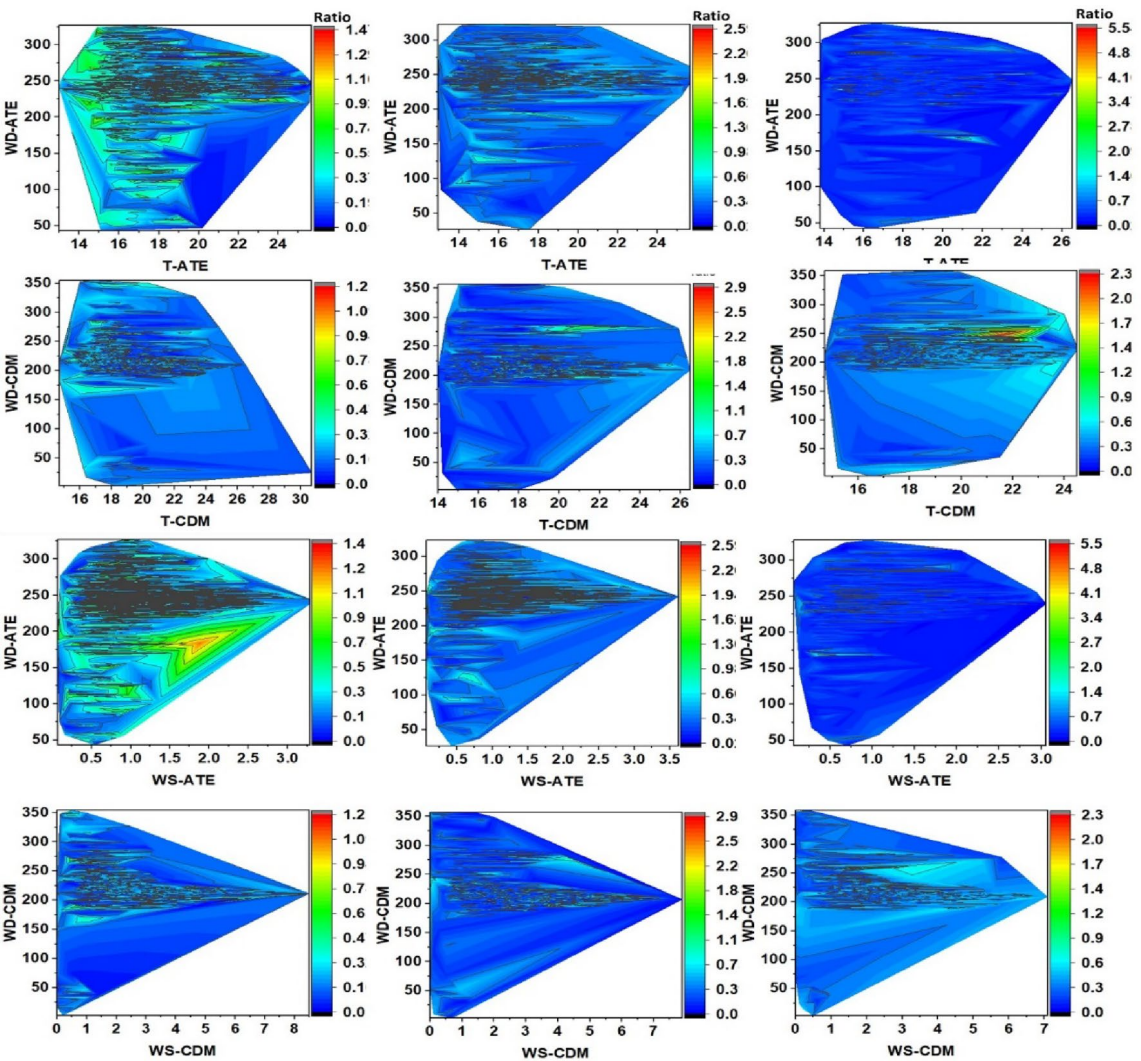
Contour lines for the distribution of (wind speed–wind direction) WD–WS-PM_2.5_/PM_10_ ratio in the spring (2016–2018) in Ate, VMT, and CDM.

**Figure 10:**
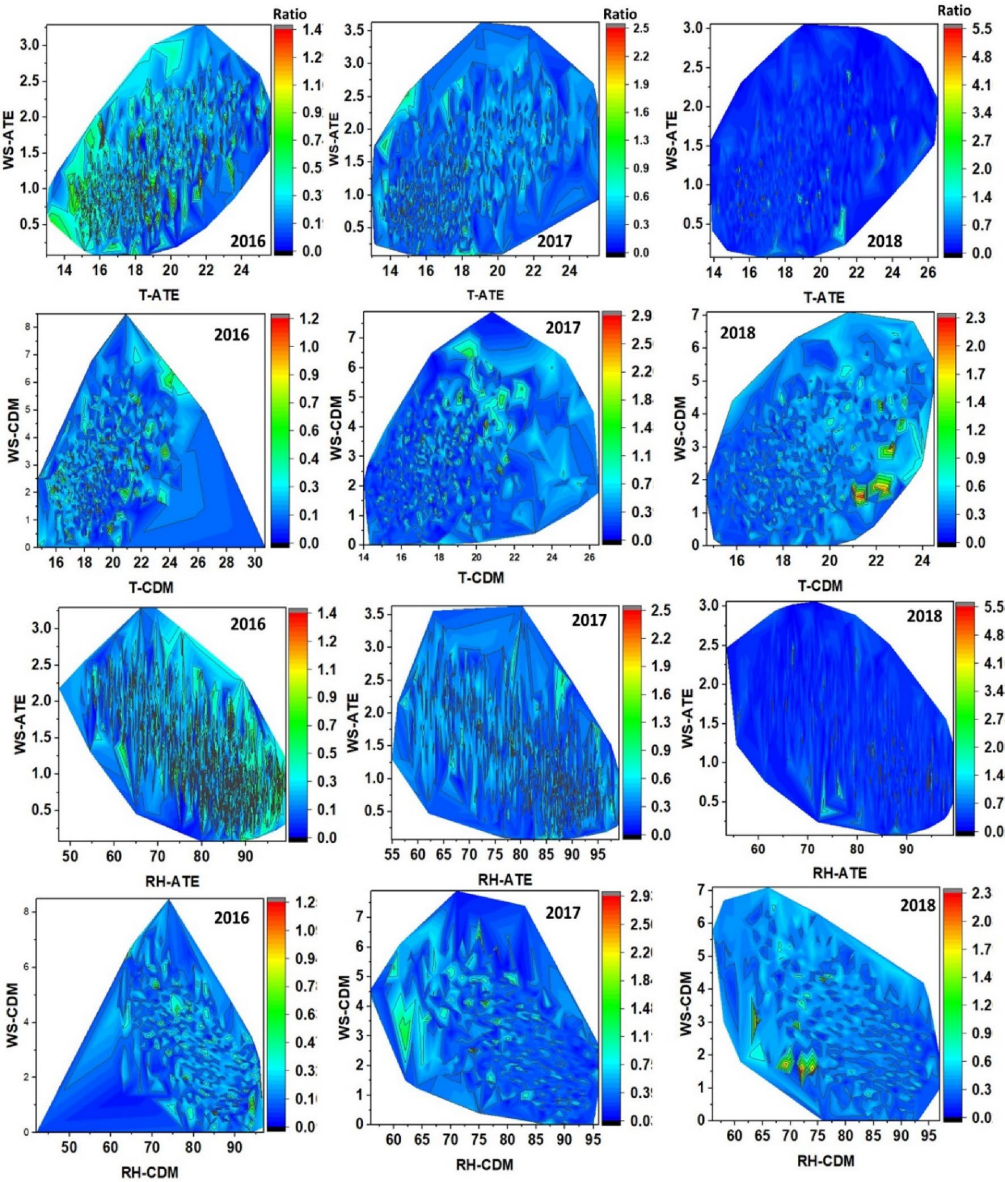
Contour lines for distribution of (wind speed–temperature) WS–T, (wind speed–relative humidity) WS–RH, and PM_2.5_/PM_10_ ratio in the austral spring (2016–2018) in Ate, VMT, and CDM.

Contour lines are shown below to represent the distribution of PM_2.5_/PM_10_ ratios associated with the climate variables. Figure 8 shows for Ate an increasing distribution between relative humidity and the PM_2.5_/PM_10_ ratio over the years between 2016 and 2018 (mean ratios: 0.33 to 0.37), but not temperature, which presented a significant decrease: in Ate (– 0.81^∘^C), in CDM (– 1.22^∘^C) and VMT (– 0.92^∘^C), developing inverse distributions between T^∘^C and HR. Figures 9 and 10 shows a dense distribution of wind direction between 200 and 250 degrees for Ate and CDM indicating the horizontal transport of air masses towards the north and northeast of Lima. Likewise, with respect to the intensity of these winds, the higher speeds dispersed the aerosols and, conversely, in more stable conditions, the PM_2.5_/PM_10_ ratios increase due to the predominance of secondary aerosols. A positive correlation of the wind speed and direction with PM_10_ indicating greater primary aerosol transport. These directions are possible sources of coarse particles. On the other hand, CDM receives lower intensity winds, favouring the transport of fine PM_2.5_ particles that reflect higher PM_2.5_/PM_10_ ratios (dominated by road traffic or other combustion sources).

### Limitations

Strong but weak correlations were not established for PM_10_, and the meteorological variables of RH and T. Likewise, no significant correlation values were obtained for PM_2.5_ with the meteorological variables. This is because this research has not considered other variables such as the topographic aspect due to the presence of two basins and hills that generate local microclimates. It is also important to highlight that in this research 90.5% of data validated by SENAMHI was used due to the absence of information on WS and WD in VMT.

## Conclusions

The concentration of airborne particles has been shown to increase during morning and evening peak hours, driven by the intensification of vehicle fleet activities and anthropogenic activities. The highest PM10 contents are observed with greater emphasis on Thursdays at 22:00 hours and decrease on Sundays, while PM_2.5_ remains practically constant throughout the week.

The dominant type of aerosol distributed in the three sampling sites has been identified using the color scatter plot method of the PM_2.5_/PM_10_ data. The contribution of the non-metallic mineral production industry stood out as one of the sources, followed by natural contributions and the resuspension of dust. Likewise, the presence of PM_2.5_ has been classified, composed of carbonaceous species from fossil fuel sources, secondary formations and biomass burning.

Weak direct correlations for temperature and inverse correlations for relative humidity have been established with PM_10_ at the local level, but no significant correlation with PM_2.5_ has been achieved. However, correlations improved for PM_10_ and PM_2.5_, as wind flows influence environmental transport, dispersion and removal of PM. The correlation of PM_10_ and PM_2.5_ with PM_2.5_/PM_10_ produced significant moderate and strong correlations respectively, which makes it possible to identify the emission sources and the levels that put the health of the inhabitants of the area at risk.

These indicators can be applied locally by local governments and can also work at a macro level, which will help optimize air quality management and control instruments.

## Data Availability

The collection and statistical processing of the data was carried out under the authorization of *Servicio Nacional de Meteorología e Hidrología del Perú*, is a specialized technical agency of the Peruvian State that provides information on weather forecasting, as well as scientific studies in the areas of hydrology, meteorology, and environmental issues. The datasets are available in the repository, https://www.senamhi.gob.pe/site/descarga-datos/.
